# Supplementary material for: Deletion of wfs1 Impairs Oligodendrocyte Precursor Cells Dorsal Distribution and Myelination Through the wfs1-hmgcs1 Axis in Zebrafish
Source: Biology (Basel). 2026 Aug 16;15(16):1402. doi: 10.3390/biology15161402 (PMC13510151; doi:10.3390/biology15161402)
Supplement: Supplementary file 1 [file biology-15-01402-s001.zip › File S1-Transcriptome sequencing data and differentially expressed genes (DEGs) in wfs1-- zebrafish at 5 dpf/2.KEGG/wfs1KOvsWT/DOWN/src/dre04210.html]

dre04210


- dre:373107

- Down regulated genes

373107\_1(-7.07514976036473)
- dre:246726

- Down regulated genes

246726(-4.22240033417505)

- dre:140621

- Down regulated genes

140621(-2.68196234303566) 140621\_1(-3.95107590777093)

- dre:58100

- Down regulated genes

58100\_1(-4.1286149197908)

- dre:373107

- Down regulated genes

373107\_1(-7.07514976036473)
- dre:246726

- Down regulated genes

246726(-4.22240033417505)

- dre:791531

- Down regulated genes

791531\_1(-4.18289781419802)
- dre:552927

- Down regulated genes

552927\_1(-4.38153959457455)

- dre:561021

- Down regulated genes

561021\_1(-4.68369619674599)

- dre:100000252

- Down regulated genes

100000252\_1(-1.7718817168434)

- dre:564295

- Down regulated genes

564295\_2(-1.27307321576804)

- dre:564295

- Down regulated genes

564295\_2(-1.27307321576804)

- dre:431763

- Down regulated genes

431763\_1(-4.20440659354098)

- dre:564295

- Down regulated genes

564295\_2(-1.27307321576804)

- dre:558237

- Down regulated genes

558237\_1(-1.94673800089826)

- dre:559564

- Down regulated genes

559564(-1.18025564761092)

- dre:57934

- Down regulated genes

57934(-1.25539807424297)

Close
